# A Rare Case of Acute Esophageal Necrosis Precipitated by Klebsiella Pneumoniae

**DOI:** 10.1016/j.gastha.2023.04.002

**Published:** 2023-04-15

**Authors:** Lekha Yadukumar, Hunain Aslam, Khalid Ahmed, Peter Iskander, Khadijah Sajid, Omar Syed, Mark M. Aloysius, Simin Nasr, Vikas Khurana

**Affiliations:** 1 Internal Medicine, The Wright Center for Graduate Medical Education, Scranton, Pennsylvania; 2 Family Medicine, The Wright Center for Graduate Medical Education, Scranton, Pennsylvania; 3 Gastroenterology, The Wright Center for Graduate Medical Education, Scranton, Pennsylvania

**Keywords:** Acute Esophageal Necrosis, Klebsiella Pneumonia, Esophagoduodenoscopy, Gastrointestinal Bleed, Coffee-ground vomitus

## Abstract

Acute esophageal necrosis is a rare condition; visualization of necrosis on esophagoduodenoscopy can help establish the diagnosis. Due to variations in blood supply, there is a higher propensity for ischemic episodes to occur along the lower esophagus; these can be of particular importance during times of hypotension. Underlying infections and atherosclerosis can further exacerbate blood supply leading to ischemia in these watershed zones. We present a case of a patient with Klebsiella pneumonia who was found to have “coffee-ground” output on nasogastric tube suctioning. Esophagoduodenoscopy was performed, which showed evidence of circumferential esophageal necrosis. Unfortunately, despite antibiotic and vasopressor support, the patient ultimately passed away.

## Introduction

Acute esophageal necrosis (AEN) is a rare ischemic condition. It is diagnosed via esophagoduodenoscopy (EGD) and is characterized by an ischemic or black appearance of the esophagus. Symptoms can be consistent with those of an upper gastrointestinal (GI) bleed, which can present as hematemesis, melena, or coffee ground emesis. AEN has a low incidence rate, up to 0.01%–0.28%.^1^ The disease is believed to be multifactorial and often associated with underlying medical comorbidities. The mainstay of treatment for AEN includes conservative medical therapy and surgical intervention for complications; prognosis is typically poor and the mortality rate is around 31.8%.^2^ We present a case of a patient found to have AEN with an etiology believed to be secondary to *Klebsiella pneumonia*. Despite antibiotic and vasopressor support, the patient's clinical course continued to deteriorate, and she eventually passed away.

## Case report

An 88-year-old male presented to the hospital after a syncopal episode. Vital signs were significant for temperature of 98 °C, heart rate of 109 beats per minute, respiratory rate of 18 breaths per minute, blood pressure of 153/93 mmHg, and oxygen saturation of 98% on room air. He was alert and oriented to person, place, and time. Physical exam was remarkable for a forehead laceration and cervical spine tenderness. Orthostatic vital signs were positive. Complete blood count (CBC) showed neutrophil-predominant leukocytosis. An electrocardiogram showed a right bundle branch block. Urinalysis was positive for hematuria. Magnetic Resonance Imaging (MRI) of the spine without contrast showed an epidural hematoma at the level of C2–C3 with a posterior cerebrospinal fluid leak. Computerized tomography (CT) of the head and spine showed global volume loss without hemorrhage and was positive for type 3 dens fracture with a bilateral cervical C6 spine fracture. Carotid duplex showed less than 50% stenosis of bilateral internal carotid arteries (ICA). CT angiogram for the neck showed 50% occlusion of the left cervical ICA, for which he was started on Aspirin 81 mg. A chest X-ray showed airspace opacities in the left lower lobe. The patient was advised compression stockings, cervical collar, and pain management as per neurosurgery. An echocardiogram showed normal left ventricular ejection fraction (55%–59%) with tricuspid regurgitation; no regional wall motion abnormalities or pericardial effusion were noted.

Infectious work up was grossly unremarkable. Pro-brain natriuretic peptide (pro-BNP) and lactate levels were elevated to 444 pg/mL (normal: < 100 pg/mL) and 1.9 mmol/L (normal: <2 mmol/L), respectively. Patient gradually started developing a productive cough, pain radiating down both arms, and difficulty with gait. Overnight telemetry showed a normal sinus rhythm with a first-degree block; heart rate was between 70–80 beats per minute with a 5-beat run of nonsustained supraventricular tachycardia and blood pressure around 150/70 mmHG. The patient was found to be hyponatremic with serum Na of 114 mEq/L (normal: 135–145 mEq/L) and serum osmolarity of 255 mosmol/kg (normal: 275–295 mosmol/kg). Mental status began to slowly deteriorate as the patient began to become more altered. Lung examination showed poor inspiratory effort and bilaterally decreased breath sounds. Later, he had an episode of “coffee-ground” emesis for which gastroenterology was consulted; a nasogastric (NG) tube was placed, and 600 mL of coffee-ground vomitus was suctioned out. An emergent EGD was performed, which revealed multiple plaques in the proximal esophagus 25 cm from the gastroesophageal junction with a circumferential “black esophagus” (Figure). Biopsies of this region were done. Further examination revealed coffee-ground material in the gastric fundus and localized circumferential erythematous mucosa was found in the gastric body. The patient was admitted to the intensive care unit for further management and started on a proton pump inhibitor infusion. Tracheal aspirate was positive for *Klebsiella pneumonia*. The patient was started on broad-spectrum antibiotic therapy consisting of vancomycin and piperacillin/tazobactam and deescalated to ceftriaxone based on culture and sensitivity. After EGD, the patient remained intubated and required transient vasopressors. Over the next few days, he had roughly 250 mL of coffee-ground output from the NG tube. Meanwhile, an EGD biopsy showed necrotic esophageal squamous mucosa with associated acute inflammation and necrotic debris. The immunostain for herpes simplex virus was negative. Later on, the patient began developing acute numbness and weakness of all extremities. MRI of the cervical spine was performed, which showed worsening posterior epidural hematoma resulting in severe spinal cord and spinal canal compression. The patient underwent an emergent laminectomy, following which he remained hypotensive, intubated, and unable to tolerate spontaneous breathing. The patient became febrile, hemodynamically unstable, hypoxic, and hypercapnic. He developed multiple further episodes of nonsustained supraventricular tachycardia and, unfortunately, passed away despite adequate measures.

**Figure:**
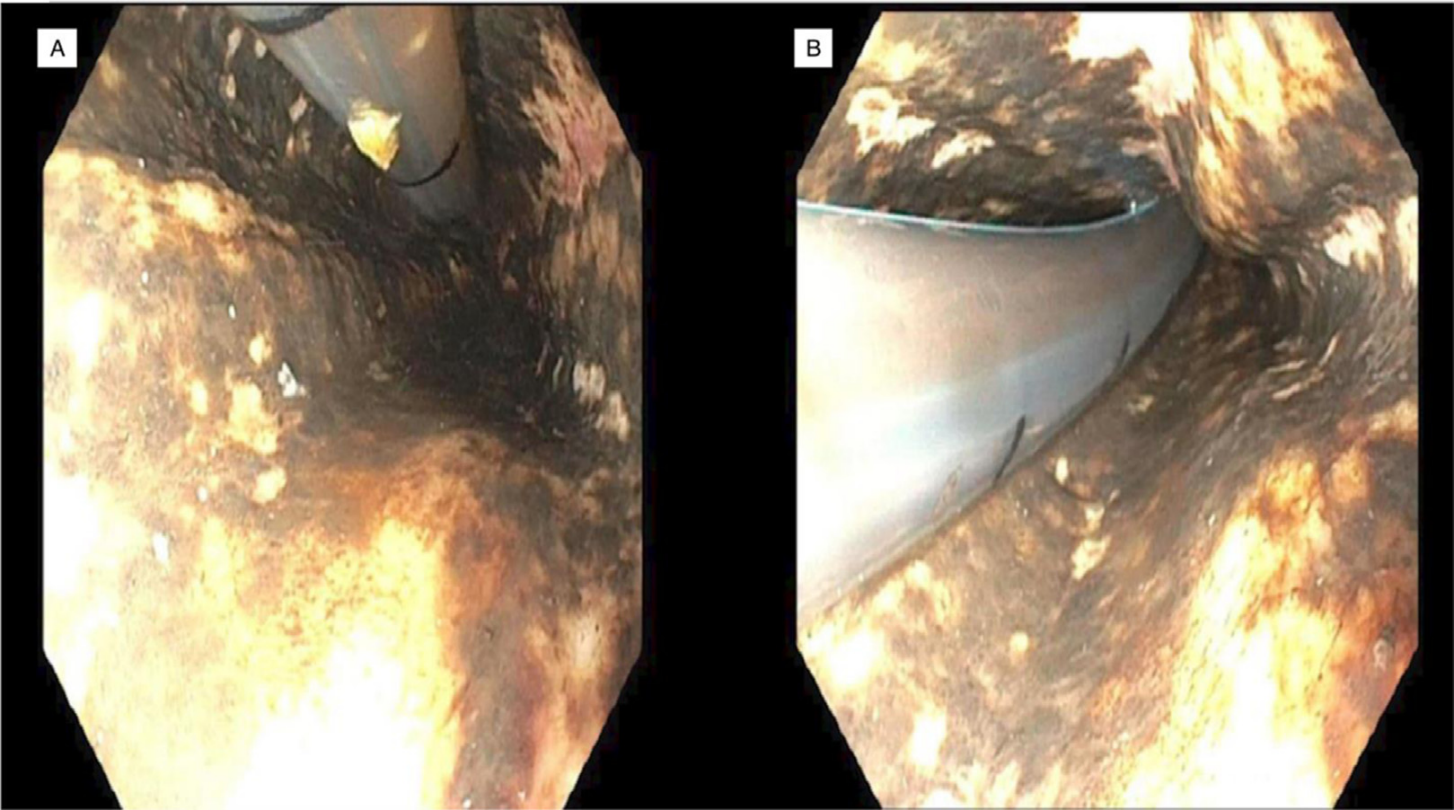
Figure depicting EGD imaging findings of patient’s “black esophagus”. (A), Figure depicting photo taken during EGD of necrosis noted around the gastroesophageal junction. (B), Figure depicting photo taken during EGD, visualizes the diffuse circumferential nature of the necrosis around the gastroesophageal junction.

## Discussion

AEN, also known as “black esophagus”, is a rare condition associated with high mortality and morbidity. It is commonly associated with an upper GI bleed and has been established as an AEN syndrome.^1^ It is diagnosed visually via EGD, showing a black-colored distal esophagus and an abrupt transition point at the gastroesophageal junction with variable proximal extension.^2^ Pathophysiology can include esophageal injury secondary to gastric reflux, hypoperfusion followed by ischemia, and weakened protective barrier.^3^, ^4^, ^5^ Many investigators suggested a “two-hit hypothesis”. The upper two-thirds of the esophagus has an abundant blood supply when compared to the lower, which has many watershed zones; this makes it susceptible to ischemia during times of hypoperfusion. According to this hypothesis, there is an initial low-flow vascular event with an altered ability to repair mucosal barrier systems (ie, malnourished or critically ill patients).^1^^,^^6^ AEN is usually found in elderly men; other risk factors include diabetes mellitus, coronary artery disease, chronic kidney disease, hypertension, etc. Other rare presentations of AEN that were reported include ischemic cholangitis, acute pancreatitis, peritonitis, and diabetic ketoacidosis.^4^^,^^7^, ^8^, ^9^, ^10^ As with our case, an epidural hematoma could have precipitated states of hypoperfusion. Furthermore, stenosis in his ICA could indicate atherosclerosis, which could also be present in the GI vasculature, further exacerbating ischemia during times of hypoperfusion.

Similar to the cases reported, our patient was noted to have *Klebsiella* pneumonia on bronchial cultures, developed coffee-ground emesis, and had EGD findings of a black discoloration of the distal esophagus.^11^, ^12^, ^13^ Commonly, AEN findings are localized to the distal one-third of the esophagus, likely due to limited vascular supply. It has been studied that the histological involvement of necrosis can spread deeply to involve the muscularis propria of the distal esophagus.^14^^,^^15^ Although a biopsy is not required for the diagnosis of AEN, it is performed to help guide management.^16^ Some studies have revealed CT angiography as a relevant tool in diagnosing AEN.^17^

The management of AEN is essentially focused on diagnosing and treating the underlying condition. It often involves fluid resuscitation to maintain adequate tissue perfusion, gastric acid suppression with proton pump inhibitor, sucralfate, and parenteral nutrition to bypass the inflamed esophagus; similarly done with our patient.^18^ Of note, an NG tube was inserted prior to the EGD. By convention, insertion of nasogastric or orogastric tubes should be avoided at all costs as the fragility of ischemic and necrosed mucosa may be complicated with perforation of the esophagus.^19^ Although our patient led a relatively active lifestyle, we believe he had significant risk factors on presentation including age, sex, atherosclerosis, hypoperfusion secondary to the hematoma, and an undiagnosed infection. All these risk factors placed the patient at a higher risk of developing AEN, which coincides with the two-hit hypothesis. Unfortunately, the patient passed away due to hemodynamic instability and respiratory failure.

Due to the complex nature of AEN’s disease pathology and the high mortality associated with it, AEN should be on the list of differential diagnoses for any upper GI bleeding. It should therefore be considered on the list of differentials, especially in patients with the risk factors of advanced vasculopathy. The pathophysiology is likely due to hypoperfusion, followed by weakened protective barrier and gastric reflux. With appropriate management, it was reported that up to 60% of patients have a favorable outcome. Regardless, the development of AEN is considered a poor prognostic factor. It has been suggested many times that the underlying health condition of a patient can be correlated with their mortality.^20^ Further studies need to be conducted to evaluate modalities for earlier diagnosis and management of AEN to improve patient outcomes.
